# Transcriptome and Metabolome Reveal Ferulic Acid as a Critical Phenylpropanoid for Drought Resistance in *Dendrobium sinense*

**DOI:** 10.3390/plants14121841

**Published:** 2025-06-15

**Authors:** Huiyan You, Ao Yi, Qiongjian Ou, Jia Wang, Jun Niu

**Affiliations:** Key Laboratory of Genetics and Germplasm Innovation of Tropical Special Forest Trees and Ornamental Plants-Ministry of Education, School of Tropical Agriculture and Forestry, Hainan University, Haikou 570228, China; wybplyx666666@163.com (H.Y.); 23220953000030@hainanu.edu.cn (A.Y.); oqj1219@163.com (Q.O.); wangjia9201@hainanu.edu.cn (J.W.)

**Keywords:** *Dendrobium sinense*, multiomics, phenylpropanoids, ferulic acid, drought stress

## Abstract

As an endemic epiphytic orchid of Hainan Island, *Dendrobium sinense* exhibits remarkable ecological and economic value, serving important ornamental and medicinal purposes. The combination of its epiphytic growth habit and the distinct dry season in Hainan (November–May) under the subtropical monsoon climate makes *D. sinense* particularly vulnerable to recurrent drought stress. Therefore, elucidating its drought tolerance mechanisms offers critical insights for both conservation strategies and stress resistance studies in *D. sinense*. Using polyethylene glycol (PEG)-induced drought stress, chlorophyll content decreased significantly with increasing PEG concentration, while MDA and proline content, SOD, POD CAT, and APX activity showed a significant increase. The analysis of physiological indicators indicated that plants have been subjected to drought stress. We then conducted the joint analysis of the metabolomics and transcriptomics data. Cluster analysis of differentially expressed genes and metabolites showed that drought stress markedly upregulates phenylpropanoid biosynthesis, with ferulic acid (FA) identified as a pivotal metabolite. Exogenous FA application alleviated drought-induced chlorophyll degradation in *D. sinense* seedlings. Heterologous expression of *DsCOMT* (a key FA biosynthetic gene) in *Arabidopsis thaliana* significantly enhanced drought survival. These results demonstrate the crucial role of FA in drought resistance and provide key insights into drought-related metabolic mechanisms.

## 1. Introduction

*Dendrobium sinense* is a perennial herb belonging to the genus *Dendrobium* of the family Orchidaceae [1]. This species grows epiphytically on cliff faces and tree trunks at elevations exceeding 1200 m [2]. From an ecological perspective, *D. sinense*, as an endemic herb on Hainan Island, contributes significantly to maintaining local ecosystem stability and biodiversity [1,3]. From an aesthetic perspective, *D. sinense* is cultivated as both potted and garden plants owing to its fragrant blooms and elegant floral architecture. From a medicinal perspective, it is a precious Chinese herbal medicine that is used for the production of health supplements, pharmaceuticals, and cosmetics [4,5]. Pharmacological studies reveal that *D. sinense* contains bioactive compounds (phenols, terpenoids, flavonoids, bibenzyls, and alkaloids) with antioxidant, anti-inflammatory, antiviral, antitumor, and anti-cataract properties [5,6,7]. Consequently, *D. sinense* possesses considerable potential for commercial development and diverse applications.

Unlike parasitic plants, epiphytes do not extract water or nutrients from their host plants; instead, they obtain these resources primarily from the surrounding environment to support their growth and development [8]. Because of a tropical monsoon climate on Hainan Island, the relative air humidity in the habitat of *D. sinense* varies significantly between seasons. *D. sinense* experiences severe drought stress in dry seasons and intermittent water deficit during rainy periods [9]. Thus, *D. sinense’s* epiphytic habit and microclimate exposure render it particularly vulnerable to recurrent drought stress. Elucidating its adaptive mechanisms will inform conservation strategies and sustainable utilization of this ecologically and economically important species. Controlled drought experiments with *D. sinense* seedlings revealed significant activation of phenylpropanoid biosynthesis under atmospheric drought stress [2].

Phenylpropanoids represent a major class of stress-induced secondary metabolites in plants, synthesized in response to both biotic and abiotic stressors [10]. The phenylpropanoid biosynthesis pathway involves complex interactions among multiple genes and interconnected metabolic networks. Both lignin and flavonoid (including anthocyanin) biosynthetic pathways utilize common phenylpropanoid-derived precursors [11]. Stress conditions regulate phenylpropanoid biosynthesis, thereby modulating metabolite accumulation [12]. These phenylpropanoids play a crucial role in plant stress response, involving the regulation of metabolic processes, osmotic adaptation, and oxidative stress [13].

Caffeic acid 3-O-methyltransferase (COMT) serves as the rate-limiting enzyme catalyzing the S-adenosylmethionine-dependent methylation of caffeic acid to ferulic acid (FA) [14]. As a pivotal phenylpropanoid metabolite, FA functions as the central precursor for coniferyl alcohol biosynthesis, determining the G/S lignin ratio in secondary cell walls. An FA deficiency reduces cuticular wax deposition and compromises epidermal integrity, accelerating moisture loss and chlorophyll degradation in *Arabidopsis thaliana* [15]. The increase in coniferyl alcohol contributes to the lignin synthesis to increase the strength and stability of cell walls in Mulberry, enabling plant cells to better withstand drought stress [16]. Tobacco studies demonstrate that enhanced FA-to-coniferyl alcohol conversion increases foliar lignin content and consequently improves drought resistance [17]. Interestingly, COMT exhibits broad substrate specificity, including 5-hydroxyferulic acid, N-acetylserotonin, caffeic aldehyde, and 5-hydroxyconiferaldehyde [18]. Overexpression of COMT1 significantly increased melatonin content in tomatoes, thereby enhancing salt tolerance in germinated seeds [19].

To further investigate phenylpropanoid pathway responses to drought, we subjected clonal tissue-cultured *D. sinense* seedlings to PEG-6000-mediated osmotic stress. We analyzed physiological parameters, transcriptomic profiles, and metabolomic changes to characterize drought responses. Importantly, this study will focus on the key phenylpropanoid metabolite of FA. Exogenous FA applications under drought conditions elucidated FA’s functional roles in *D. sinense* stress adaptation. The COMT function was investigated through heterologous expression studies in *A. thaliana*. The study provides us with a deeper understanding of molecular mechanisms and pathways operating in the drought tolerance of *D. sinense*.

## 2. Results

### 2.1. Physiological and Biochemical Indicators Related to Drought Stress

**Figure 1:**
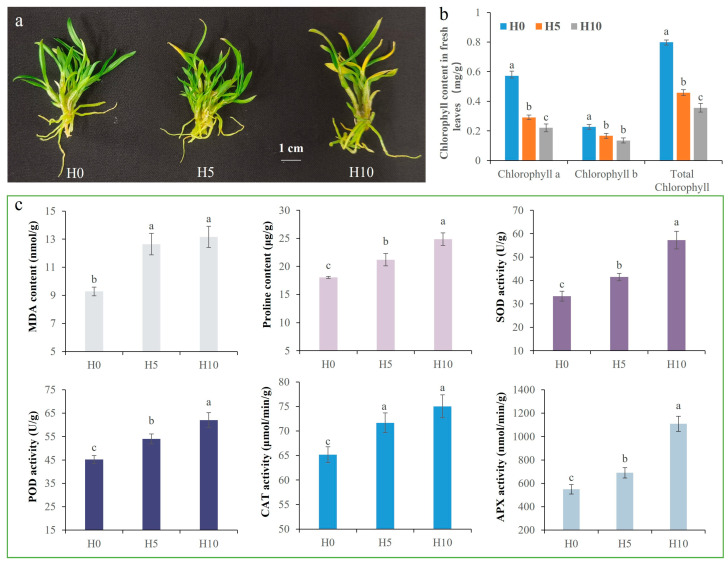
Physiological responses of *D. sinense* to PEG-6000-induced drought stress. (**a**) Phenotypic manifestations after 20 days of treatment at 0% (H0), 5% (H5), and 10% (H10) PEG-6000, showing progressive leaf chlorosis with increasing stress intensity. (**b**) Significant reduction in chlorophyll content (*p* < 0.05) across stress gradients in fresh leaves. (**c**) Effects of drought stress on physiological and biochemical parameters of fresh leaves. Values represent mean ± SE (*n* = 3 biological replicates). Distinct lowercase letters indicate statistically significant differences (*p* < 0.05).

The PEG-induced osmotic stress, including 0% (H0), 5% (H5), and 10% (H10) PEG-6000, was used to simulate drought stress in tissue culture seedlings of *D. sinense*. After 20 days of treatment, H10-group plants exhibited severe leaf chlorosis, while H5-group plants showed moderate chlorosis compared to H0 controls (Figure 1a). As expected, chlorophyll content in fresh leaves decreased significantly with increasing PEG concentration (Figure 1b). Leaf malondialdehyde (MDA) content was significantly elevated in both H5 and H10 treatments compared to unstressed controls (Figure 1c). To maintain redox homeostasis, the proline (PRO) content, superoxide dismutase (SOD) activity, peroxidase (POD) activity, catalase (CAT) activity, and ascorbate peroxidase (APX) activity showed a scheduled and significant increase as the drought intensified (Figure 1c). These findings confirm successful induction of drought stress and validate the physiological responsiveness of *D. sinense* seedlings.

### 2.2. Identification of Metabolites Responsive to Drought Stress

**Figure 2:**
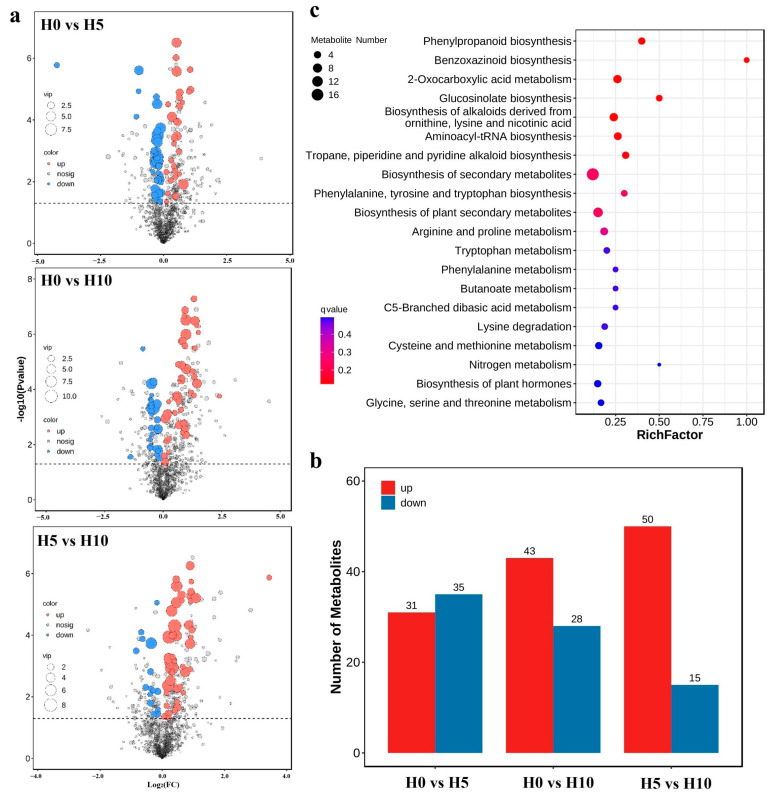
Metabolomic profiling of *D. sinense* under PEG-induced drought stress. (**a**) Volcano plot analysis of differential metabolites. (**b**) Quantitative distribution of significantly altered metabolites across treatment comparisons (H0 vs. H5, H0 vs. H10, H5 vs. H10). (**c**) KEGG pathway enrichment analysis showing top significantly affected metabolic pathways.

Metabolomic profiling identified 950 metabolites in *D. sinense*, classified into 20 chemical categories. Nucleotides and derivatives (*n* = 187, 19.68%) and flavonoids (*n* = 127, 13.37%) represented the most abundant metabolite classes (Supplementary Table S1). The assessment of correlation between samples was performed using the Pearson correlation coefficient algorithm. Compared with intergroup samples, intragroup samples showed a higher correlation (Supplementary Figure S1). Pairwise comparative analysis revealed significant metabolite alterations across treatment groups (Figure 2a). In total, 31 upregulated and 35 downregulated differential metabolites were identified in H0 VS H5 group, 43 upregulated and 28 downregulated in H0 VS H10 group, 50 upregulated and 15 downregulated in H5 VS H10 group (Figure 2b). To further investigate the biological functions of these differential metabolites, pathway enrichment analysis was performed. The metabolic changes mainly involved phenylpropanoid biosynthesis (ko00940), benzoxazinoid biosynthesis (ko00402), 2-oxocarboxylic acid metabolism (ko01210), and glucosinolate biosynthesis (ko00966) (Figure 2c).

### 2.3. Transcriptome Analysis Results

**Figure 3:**
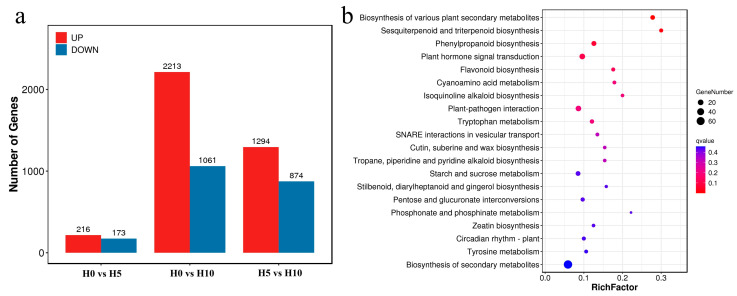
Transcriptomic analysis of DEGs in response to drought stress. (**a**) Quantitative distribution of DEGs across distinct experimental groups. (**b**) KEGG pathway enrichment analysis of DEGs.

Illumina sequencing generated 422,148,522 high-quality clean reads. The raw sequencing data have been deposited in the National Genomics Data Center (BioProject accession: PRJCA039371). Assembly of these reads yielded 71,063 unigenes (mean length = 1007 bp). Functional annotation was achieved for 32,982 unigenes in at least one reference database. Gene expression levels were quantified using fragments per kilobase of transcript per million mapped reads (FPKM). Subsequently, we identified differentially expressed genes (DEGs) responsive to drought stress. Comparative analysis revealed 389 DEGs between H0 and H5 (216 upregulated and 173 downregulated), 3274 DEGs between H0 and H10 (2213 upregulated and 1061 downregulated), and 2168 DEGs between H5 and H10 (1294 upregulated and 874 downregulated) (Figure 3a). To further elucidate the biological functions of DEGs, we conducted pathway enrichment analysis. The most significantly enriched pathways included biosynthesis of various plant secondary metabolites (ko00999), sesquiterpenoid and triterpenoid biosynthesis (ko00909), and phenylpropanoid biosynthesis (ko00940) (Figure 3b).

### 2.4. The Response of Phenylpropanoid Biosynthesis to Drought Stress

**Figure 4:**
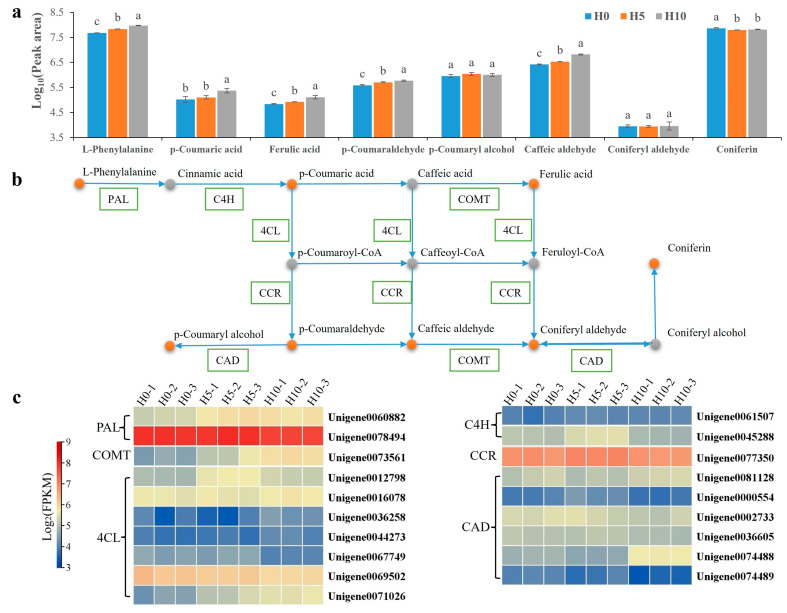
The phenylpropanoid biosynthetic pathway is responsive to drought stress. (**a**) The upper bar chart displays the phenylpropanoid contents. Lowercase letters indicate significant differences (different letters indicate *p* < 0.05). (**b**) The middle diagram illustrates the primary phenylpropanoid biosynthesis pathway in response to drought stress. Orange dots indicate the presence of the metabolite, whereas gray dots indicate its absence. (**c**) The heatmap below illustrates the expression profiles of the associated genes.

Integrated transcriptomic and metabolomic analyses demonstrated phenylpropanoid biosynthesis as a central drought-responsive pathway. The primary metabolic pathway of L-phenylalanine in *D. sinense* was constructed. Cytosolic phenylalanine ammonia-lyase (PAL) initiates phenylpropanoid biosynthesis by converting L-phenylalanine to cinnamic acid. L-phenylalanine accumulation increased progressively with drought severity (1.43-fold at H5, 1.97-fold at H10, Figure 4a,b). Among the two identified *PAL* genes, Unigene0060882 showed drought-inducible expression (1.47-fold at H5, 1.46-fold at H10), while Unigene0078494 maintained constitutive and high expression (Figure 4c). The pathway proceeds via C4H-mediated hydroxylation to p-coumaric acid, followed by COMT-catalyzed methylation to ferulic acid (Figure 4b). The p-coumaric acid content was significantly elevated only in the H10 treatment group (2.22-fold), while FA levels increased progressively (1.22-fold at H5, 1.91-fold at H10) (Figure 4a). Two *C4H* genes displayed differential regulation: Unigene0061507 maintained stable expression, while Unigene0045288 peaked at H5 group (1.34-fold increase) (Figure 4c). The expression levels of *D. sinense COMT* (*DsCOMT*; Unigene0073561) increased progressively with drought severity (1.70-fold at H5, 2.53-fold at H10) (Figure 4c). Our previous atmospheric drought experiments established a consistent positive correlation between drought intensity, *DsCOMT* expression, and FA accumulation [2]. These results demonstrate that drought-induced FA biosynthesis constitutes a key metabolic adaptation for drought resistance in *D. sinense*.

Downstream phenylpropanoid derivatives were synthesized via 4-coumarate-CoA ligase (4CL) and cinnamoyl-CoA reductase (CCR) activities (Figure 4b). Although these *4CL* genes displayed differential expression patterns under drought stress, only Unigene0071026 expression increased progressively with drought severity (1.66-fold at H5, 2.13-fold at H10, Figure 4c). The single *CCR* gene maintained constitutively high expression (FPKM 115.68 ± 8.35 across treatments) regardless of drought stress (Figure 4c). Cinnamyl-alcohol dehydrogenase (CAD) catalyzes the NADPH-dependent reduction in hydroxycinnamaldehydes to monolignols, the immediate precursors for lignin biosynthesis. Among six CAD genes, Unigene0074488 (peak 1.80-fold at H10), while others responded variably. p-coumaryl alcohol showed stable accumulation, whereas coniferin content showed 85.27% and 89.72% reduction at H5 and H10, respectively (Figure 4a).

### 2.5. Alleviating Effect of Ferulic Acid on Drought Stress of D. sinense

**Figure 5:**
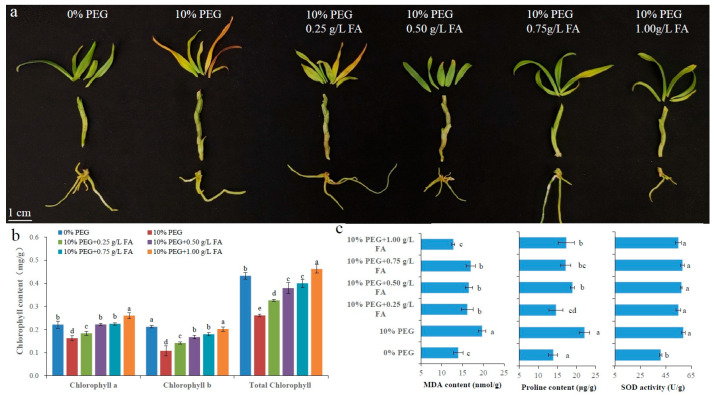
Alleviating effect of FA on drought stress in *D. sinense.* (**a**) Phenotypic observation of *D. sinense* under different concentrations of FA treatment. (**b**) The results of chlorophyll content determination in fresh *D. sinense* leaves. Lowercase letters indicate significant differences (different letters indicate *p* < 0.05). (**c**) MDA, PRO, and SOD levels in fresh *D. sinense* leaves. Lowercase letters indicate significant differences (different letters indicate *p* < 0.05).

To evaluate the role of FA in enhancing drought tolerance in *D. sinense*, plants were treated with different concentrations of FA in the culture medium. After 20 days of cultivation, the 10% PEG treatment caused severe leaf yellowing, wilting, shedding, and even plant death compared to the control (0% PEG) (Figure 5a). Leaves still exhibited noticeable yellowing in the 10% PEG + 0.25 g/L FA treatment, but yellowing was significantly alleviated in the other three FA treatment groups (Figure 5a). Chlorophyll analysis revealed a significant increase in *D. sinense* leaves with FA treatment, peaking in the 10% PEG + 1.00 g/L FA group (Figure 5b). These results suggest that exogenous FA could prevent the chlorophyll degradation in *D. sinense* leaves. Notably, PEG + FA treatment significantly reduced MDA and PRO levels compared to 10% PEG alone, with the lowest levels observed in the 1.00 g/L and 0.25 g/L FA treatment groups, respectively (Figure 5c). However, FA treatment did not affect SOD activity under drought stress (Figure 5c). Collectively, exogenous FA enhances drought stress resistance in *D. sinense*.

### 2.6. Heterologous Expression of DsCOMT in A. thaliana

**Figure 6:**
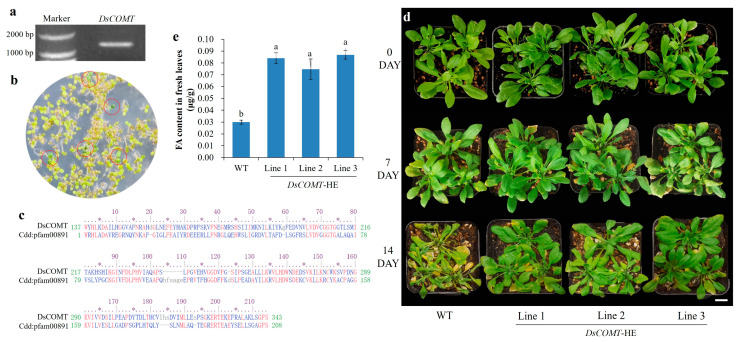
Heterologous expression of *DsCOMT* in *A. thaliana*. (**a**) Image of agarose gel electrophoresis from *DsCOMT* amplification. (**b**) The analysis of conserved protein domain families. The analysis of the Conserved Domain Database showed that DsCOMT protein contained the SAM domain (pfam00891). The first line is the DsCOMT protein sequence, and the second line is the SAM domain sequence. (**c**) Screening diagram of resistant plants. Red circles indicated that the seedlings exhibited resistance to kanamycin. (**d**) Comparison between wild-type and transgenic *Arabidopsis* plants under drought stress treatment. Each pot contains three plants. One-month-old wild-type and transgenic plants were subjected to drought stress by withholding watering for 0, 7, and 14 days. *DsCOMT*-HE represents the heterologous expression of *DsCOMT* in wild-type *Arabidopsis*. Scale bar in the right lower picture: 1 cm. (**e**) HPLC analysis of FA contents in fresh leaves from transgenic and wild-type plants. To meet the required sample amount for the analysis, all leaves from the *A. thaliana* plants in the same pot were collected. Lowercase letters indicate significant differences (different letters indicate *p* < 0.05).

To elucidate the role of FA and its associated *DsCOMT* gene in drought response, we cloned and sequenced the full-length coding region of *DsCOMT* (Figure 6a). The open reading frame (ORF) spanned 1086 bp, encoding a 361-amino-acid protein. The analysis of conserved protein domain family revealed that DsCOMT protein belongs to the family of S-adenosylmethionine-dependent methyltransferases (SAM) (Figure 6b). The *DsCOMT* gene was transferred into the pBI121 eukaryotic expression vector. Transgenic *A. thaliana* lines were generated using the floral dip method with Agrobacterium-mediated transformation. Four stable transgenic *A. thaliana* lines were verified by antibiotic resistance screening and PCR analysis (Figure 6c and Supplementary Figure S2).

Under normal growth conditions, transgenic and wild-type *A. thaliana* showed no phenotypic differences after one month of cultivation, including rosette leaf diameter, leaf number, and leaf length and width (Figure 6d and Supplementary Table S2). Rosette leaves from one-month-old *DsCOMT*-expressing transgenic plants were harvested for FA quantification via high-performance liquid chromatography (HPLC). *DsCOMT*-expressing plants accumulated approximately 4-fold higher FA levels compared to wild-type controls (Figure 6e). Following 7 days of water deprivation, transgenic and wild-type plants exhibited mild leaf yellowing (Figure 6d). After 14 days of drought stress, wild-type *A. thaliana* showed severe yellowing and mortality, whereas *DsCOMT*-transgenic plants maintained only mild chlorosis (Figure 6d). Taken together, these findings demonstrate that heterologous *DsCOMT* expression enhances drought tolerance in *Arabidopsis* plants.

## 3. Discussion

*D. sinense* is a perennial epiphytic herb belonging to the genus *Dendrobium* of the family Orchidaceae, which has been valued for centuries as both an ornamental and medicinal plant [7]. Unlike terrestrial plants, epiphytes lack subterranean growth structures. Consequently, this species depends entirely on water absorption from its host substrates (branches and rocks), rendering it particularly vulnerable to drought stress [20]. Hainan Island has a subtropical monsoon climate, with obvious dry and wet seasons [9]. The ecological challenges faced by *D. sinense* are further compounded by the climatic conditions of its natural habitat. Consequently, water availability represents the primary limiting factor for epiphytic orchid physiology in this region. Elucidating drought response mechanisms of *D. sinense* is crucial for conservation strategies and sustainable utilization of this valuable species.

Plant drought stress responses involve complex physiological processes, including metabolic reprogramming, signal transduction, osmoregulation, and transcriptional regulation [21]. Epiphytic leaves typically exhibit greater succulence and drought tolerance compared to terrestrial plants [22]. Epiphytic orchids commonly develop adaptive morphological features, including fleshy pseudobulbs and coriaceous leaves [23]. Moreover, most epiphytic orchids utilize crassulacean acid metabolism (CAM), an adaptive photosynthetic pathway that enhances water-use efficiency [2,24]. Current research has mainly investigated epiphytic orchid drought adaptations through morphological and physiological approaches, particularly water-balance strategies and leaf hydraulics [23,25]. However, secondary metabolic responses and molecular regulatory mechanisms underlying drought adaptation remain poorly characterized in *Dendrobium* species.

To identify key secondary metabolic pathways in *D. sinense* under drought stress, we previously conducted drought stress experiments under varying air humidity conditions. Integrated transcriptomic and metabolomic analyses revealed phenylpropanoid biosynthesis as a dominant drought-responsive pathway in *D. sinense* [2]. These findings align with our current results, demonstrating drought-induced activation of phenylpropanoid biosynthesis (Figure 3 and Figure 4). Notably, both FA accumulation and *DsCOMT* expression were upregulated under air drought stress and PEG-simulated drought conditions (Figure 5) [2]. Additionally, FA was considered a key metabolite for rice [26], triticale [27], and *A. thaliana* [15] drought tolerance. Therefore, we hypothesize that FA contributes significantly to drought stress adaptation.

The plant phenylpropanoid pathway forms a complex metabolic network that serves as the primary biosynthetic route for key secondary metabolites, including phenolic compounds, flavonoids, anthocyanins, and lignins [28]. These metabolites participate in diverse physiological processes, particularly in plant responses to biotic and abiotic stresses [29]. In *A. thaliana*, reduced FA levels decrease cuticular wax deposition, compromising the epidermal barrier and accelerating leaf water loss and chlorophyll degradation [15]. It was reported that FA protects photosynthetic apparatus from drought-induced damage in triticale [27]. Consistent with this, FA supplementation (PEG + FA) in *D. sinense* maintained higher chlorophyll levels compared to PEG treatment alone (Figure 5b). Notably, 1.00 g/L FA treatment in *D. sinense* restored chlorophyll content to control levels (Figure 5b). While drought stress markedly reduced chlorophyll content in *D. sinense* (Figure 1b), exogenous FA application effectively mitigated this reduction (Figure 5b). These findings suggest that exogenous FA protects *D. sinense* from drought-induced chlorophyll degradation.

We identified the key functional gene involved in FA biosynthesis. Conserved domain analysis revealed that DsCOMT belongs to the SAM-dependent methyltransferase family, which mediates crucial biocatalytic methylation reactions in diverse metabolic pathways [30]. Heterologous *DsCOMT* expression in *A. thaliana* significantly enhanced leaf FA accumulation (Figure 6e). This finding aligns with established COMT functions in FA biosynthesis [18], confirming its catalytic role. Notably, *DsCOMT*-expressing plants exhibited markedly improved drought tolerance compared to wild-type controls (Figure 6d). *A. thaliana* expressing *DsCOMT* showed enhanced drought stress tolerance. Based on the previous FA treatment experiment, it is reasonable to speculate that DsCOMT catalyzes the accumulation of FA synthesis, preventing the loss of chlorophyll, thereby enhancing the plant’s drought resistance. However, the underlying molecular mechanisms warrant further investigation.

Given that some phenylpropanoid pathway products act as precursors for other pathways, stimulating this pathway may affect downstream pathways, including the lignin and flavonoid pathways [31]. Lignin promotes cell wall synthesis and construction, thereby enhancing cell wall strength and stability, which helps maintain plant cell resistance to drought stress [16]. Additionally, flavonoids play a major role in plant responses to both biotic and abiotic stresses [32], including antioxidant defense [33], metabolic regulation, and signal transduction [34]. As an important intermediate product of secondary metabolism, the drought resistance mechanism mediated by FA may be more complex than previously thought. Moreover, COMT, as a member of the SAM family, may catalyze the methylation of other substrates. For example, COMT in *Carex rigescens* can regulate melatonin synthesis, which, as an important plant regulatory factor, enhances antioxidant capacity and stress tolerance in tobacco [35]. Collectively, the drought resistance mechanism mediated by FA biosynthesis is likely to be relatively conserved, though the specific mechanisms may be extremely complex and require further investigation.

## 4. Material and Methods

### 4.1. Experimental Materials and Drought Treatment

The clonal tissue-culture seedlings of *D. sinense* were grown in the tissue culture room, Hainan University, DanZhou, Hainan Province, China. For the drought stress treatment, uniformly growing one-year-old aseptic seedlings were selected. Based on previous PEG-simulated drought treatment in *Dendrobium* plants [36], 0%, 5%, and 10% PEG-6000 were added to the 1/2MS medium and were marked as H0, H5, and H10, respectively. Three biological replicates were performed in triplicate for each treatment. On the 20th day after treatment, significant phenotypic changes (the leaves turn yellow and the plants dwarf) are observed. To explore the FA roles in drought stress, 0.25 g/L, 0.50 g/L, 0.75 g/L, and 1.00 g/L FA were respectively added to the H10 treatment group. Subsequently, drought stress treatment was applied for the same duration. The leaf samples were quickly frozen in liquid nitrogen after cleaning with sterile water and stored at −80 °C until use.

### 4.2. Physiological and Biochemical Index Determination

For each determination, 0.1 g of fresh leaf is weighed and ground into powder. The extraction steps and standardized activity should be operated according to the instructions, including Chlorophyll content assay kit (item number: CPL-2-G, COMIN, Suzhou, China), MDA content measurement kit (item number: MDA-2-Y, COMIN, Suzhou, China), SOD activity assay kit (item number: SOD-2-W, COMIN, Suzhou, China), POD activity assay kit (item number: POD-2-Y, COMIN, Suzhou, China), CAT activity assay kit (item number: CAT-2-W, COMIN, Suzhou, China), APX activity assay kit (item number: APX-2-W, COMIN, Suzhou, China), and proline content assay kit (item number: G0111F COMIN, Suzhou, China).

### 4.3. Metabolome Analysis

The 100 mg fresh leaf was individually ground in liquid nitrogen, and the homogenate was resuspended with pre-chilled 80% methanol and 0.1% formic acid by vortexing well. The samples were incubated on ice for 5 min and then were centrifuged at 15,000× *g*, 4 for 20 min. Some of the supernatant was diluted to a final concentration containing 53% methanol by LC-MS grade water. The samples were subsequently transferred to a fresh Eppendorf tube and then were centrifuged at 15,000× *g*, 4 °C for 20 min. Finally, the supernatant was injected into the LC-MS/MS system for analysis. The analysis included three biological replicates.

Metabolomic profiling was performed in collaboration with Gene Denovo Biotechnology Co., Ltd. (Guangzhou, China). LC-MS/MS analyses were performed using an ExionLC™ A D system (SCIEX) coupled with a QTRAP^®^ 6500+ mass spectrometer (SCIEX) in Genedenovo, Guangzhou, China). Samples were injected onto an Xselect HSS T3 (2.1 × 150 mm, 2.5 μm) using a 20 min linear gradient at a flow rate of 0.4 mL/min for the positive/negative polarity mode. The eluents were eluent A (0.1% Formic acid water) and eluent B (0.1% Formic acid acetonitrile). The solvent gradient was set as follows: 2% B, 2 min; 2 100% B, 15.0 min; 100% B, 17.0 min 100 2% B, 17.1 min 2% B, 20 min. QTRAP^®^ 6500+ mass spectrometer was operated in positive polarity mode with Curtain Gas of 35 psi, Collision Gas of Medium, Ion Spray Voltage of 5500V, Temperature of 550 °C, Ion Source Gas of 1 60, Ion Source Gas of 2 60. QTRAP^®^ 6500+ mass spectrometer was operated in negative polarity mode with Curtain Gas of 35 psi, Collision Gas of Medium, Ion Spray Voltage of 4500V, Temperature of 550 °C, Ion Source Gas of 1 60, Ion Source Gas of 2 60.

### 4.4. Metabolites Identification and Quantification

The detection of the experimental samples using MRM (Multiple Reaction Monitoring) were based on a house database (GENE DENOVO, Guangzhou, China). The Q3 were used to the metabolite quantification. The Q1, Q3, RT (retention time), DP (declustering potential) and CE (collision energy) were used to the metabolite identification. The data files generated by HPLC-MS/MS were processed using the SCIEX OS Version 1.4 to integrate and correct the peak. The main parameters were set as follows: minimum peak height, 500; signal/noise ratio, 5; gaussian smooth width, 1. The area of each peak represents the relative content of the corresponding substance. We combined the multivariate statistical analysis of the VIP value of OPLS-DA (VIP ≥ 1) and the univariate statistical analysis of the t -test *p* -value (*p* < 0.05) to screen differential metabolites among different comparison groups [37].

### 4.5. RNA Extraction, Library Construction, and Sequencing

Total RNA was extracted using Trizol reagent kit (Invitrogen, Carlsbad, CA, USA) according to the manufacturer’s protocol. RNA quality was assessed on an Agilent 2100 Bioanalyzer (Agilent Technologies, Palo Alto, CA, USA) and checked using RNase free agarose gel electrophoresis. After total RNA was extracted, eukaryotic mRNA was enriched by Oligo(dT) beads. Then the enriched mRNA was fragmented into short fragments using fragmentation buffer and reverse transcribed into cDNA by using NEBNext Ultra RNA Library Prep Kit for Illumina (NEB #7530, New England Biolabs, Ipswich, MA, USA). The purified double-stranded cDNA fragments were end-repaired, a base was added, and they were ligated to Illumina sequencing adapters. The ligation reaction was purified with the AMPure XP Beads (1.0X), and amplified by polymerase chain reaction (PCR). The resulting cDNA library was sequenced using Illumina Novaseq6000 by Gene Denovo Biotechnology Co. (Guangzhou, China).

### 4.6. Gene Expression Profiles

The mapped reads of each sample were assembled by using StringTie v1.3.1 [38]. For each transcription region, the value of fragment per kilobase of transcript per million mapped reads (FPKM) was calculated to quantify its expression abundance and variations, using RSEM v1.20.0 [39]. RNA differential expression analysis was performed by DESeq2 v1.20.0 between the two different groups [40]. The genes with the parameter of false discovery rate (FDR) below 0.05 and absolute fold change ≥2 were considered differentially expressed genes/transcripts.

### 4.7. Genetic Transformation and Drought Treatment in A. thaliana

The cloning of *COMT* genes was achieved using the primers COMT1-S and COMT1-A (Supplementary Table S3). The resulting PCR products were then cloned into PCloneEZ-TOPO (Solarbio, Beijing, China), and the sequencing was carried out by Sangon, Guangzhou, China. The linearization of the pBI121 vector was conducted by *Xba*I and *Bam*HI (Tolobio, Beijing, China). Using the special primers of pBI121-*COMT*-S and pBI121-*COMT*-A (Supplementary Table S3), the *COMT1* genes were seamlessly integrated into the pBI121 to produce pBI121-COMT1 by ClonExpress^®^ II One Step Cloning Kit (Vazyme, Nanjing, China). The pBI121-COMT1 vector was transformed into *Agrobacterium tumefaciens* GV3101. The floral dip method was used for genetic transformation in *A. thaliana*, and the T3 homozygous transgenic lines were screened as previously described [41]. Briefly, *A. tumefaciens* strain GV3101 harboring the pBI121-COMT1 vector was cultured overnight, resuspended in infiltration medium (5% sucrose, 0.05% Silwet L-77), and used to dip flowering *A. thaliana* plants for 30 s. After 24 h of dark incubation, treated plants were grown to maturity under standard conditions (22 °C; 120 μmol/m^2^/s light intensity; 16 h light/8 h dark cycle; 60% relative humidity). T1 seeds were surface-sterilized and selected on MS medium containing kanamycin. Resistant T1 plants were self-pollinated to generate T2 progeny. The primers G418-S and G418-A (Supplementary Table S3) were used for PCR screening that confirmed transgene integration. T3 homozygous lines were obtained by repeating selection and genotyping in the T2 generation, ensuring stable inheritance (≥3:1 Mendelian ratio in T2).

Arabidopsis was cultivated in a plant incubator (22 °C; 120 μmol/m^2^/s light intensity; 16 h light/8 h dark cycle; 60% relative humidity). One-month-old plants with uniform growth status were used for drought treatment. Phenotypic changes were observed at 0, 7, and 14 days after stopping watering. Leaf samples were collected after 14 days of drought application and used for biochemical index determination.

### 4.8. HPLC Analysis of Ferulic Acid Content

The 0.20 g of fresh leaves was ground into fine powder with liquid nitrogen. After dissolution in 5 mL of anhydrous ethanol, a 15-min ultrasonic treatment was performed. After centrifugation at 12,000 rpm for 5 min, the extracting solution was collected and filtered through a 0.22 μm filter membrane. Then, samples were transferred to a 10 mL volumetric flask and diluted to the mark with anhydrous ethanol. A 20 μL solution was subjected to analysis by high-performance liquid chromatography (HPLC).

The samples were detected by LC-100 pump (Wufeng, Shanghai, China) with ZORBAX SB-C18 (5 μm, 4.6 × 250 mm). Chromatographic conditions: column temperature 35 °C; sample volume 20 μL, solution A 100% acetonitrile; solvent B 0.05% phosphate water; flow rate 1 mL/min. Setting the mobile phase gradient: 0–4 min, solution A 3%, solution B 97%; 4–10 min, solution A 3–80%, solvent b 97–20%; 10–15 min solution A 80%, solvent B 20%; 15–20 min, solution A 80–3%, solvent b 20–97%; 20–25 min solution A 3%, solvent B 97%. The standard substance of ferulic acid (HPLC ≥ 98%) was purchased from Solarbio life Sciences, Beijing, China.

## 5. Conclusions

This study demonstrates that FA plays a critical role in enhancing drought resistance in *D. sinense*. Under drought stress, the phenylpropanoid biosynthesis pathway was significantly activated, leading to increased FA accumulation. Exogenous FA application alleviated drought-induced physiological damage, such as chlorophyll degradation. Heterologous expression of *DsCOMT* in *A. thaliana* improved drought tolerance by elevating FA levels. These results underscore the importance of FA in plant stress responses and suggest its potential as a target for breeding drought-resistant cultivars. Further research is needed to explore the broader regulatory mechanisms of FA in stress adaptation and its applications in agricultural and ecological conservation efforts.

## Data Availability

The raw sequencing data was deposited in the National Genomics Data Center, BioProject accession: PRJCA039371.

## Supplementary Materials

The following supporting information can be downloaded at: https://www.mdpi.com/article/10.3390/plants14121841/s1. Supplementary Figure S1: Pearson correlation coefficient of metabolomic samples. Supplementary Figure S2: PCR assays of transgenic *Arabidopsis* plants. Supplementary Table S1: Metabolomic analysis of *Dendrobium sinense* under different degrees of drought stress. Supplementary Table S2: Phenotypes of wild-type and transgenic lines. Supplementary Table S3: The primers for gene cloning and vector construction.
