# Supplementary material for: Transcriptome and Metabolome Reveal Ferulic Acid as a Critical Phenylpropanoid for Drought Resistance in Dendrobium sinense
Source: Plants (Basel). 2025 Jun 15;14(12):1841. doi: 10.3390/plants14121841 (PMC12197015; doi:10.3390/plants14121841)
Supplement: Supplementary file 1 [file plants-14-01841-s001.zip › Supplementary Figures.pdf]

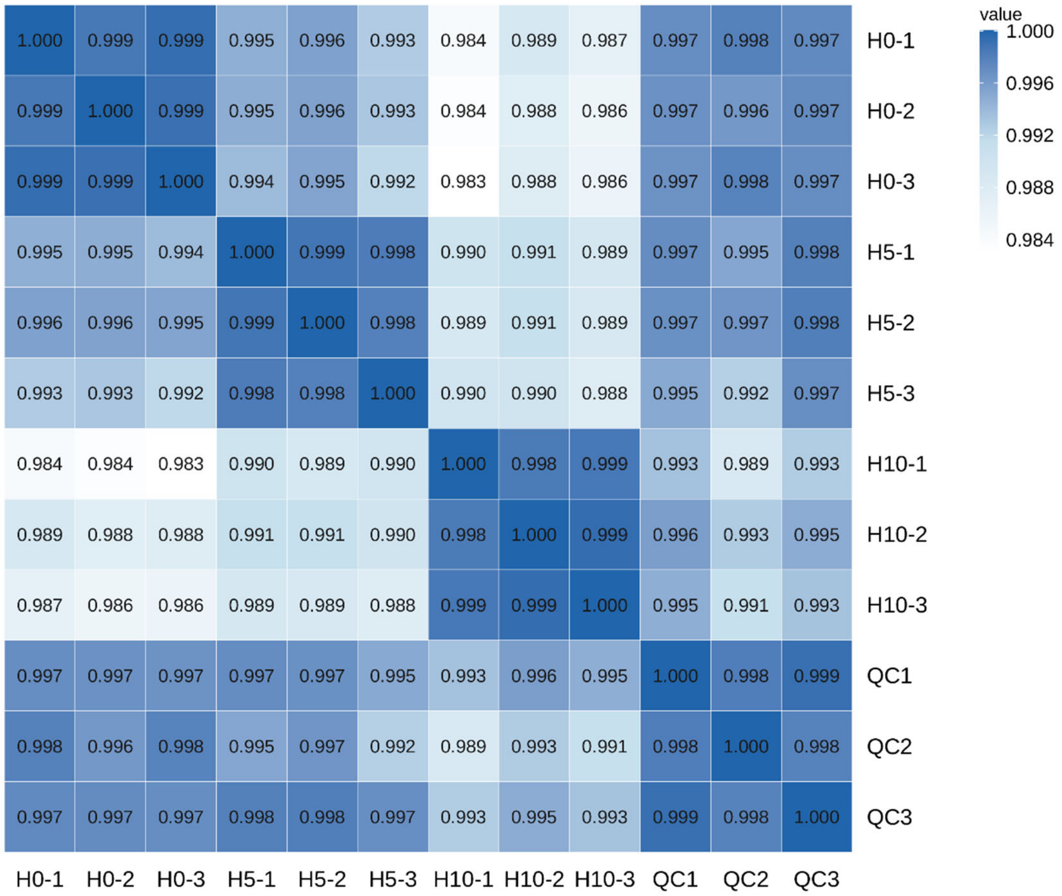

Figure S1 Pearson correlation coefficient of metabolomic samples. The greater the value, the greater the correlation.

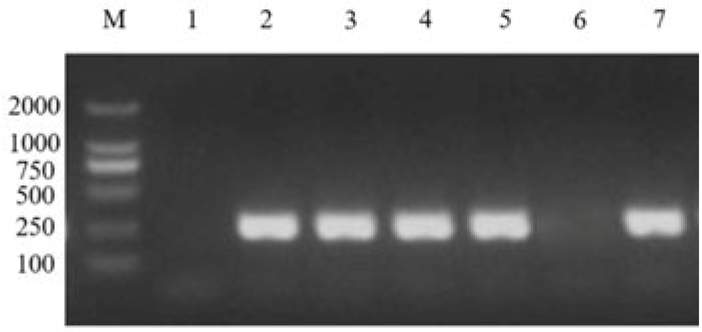

Figure S2 PCR assays of transgenic *Arabidopsis* plants.

M: 2,000 bp maker; 1: Wild-type; 2-6: transgenic *Arabidopsis*; 6: Positive control
