# Supplementary material for: Transcriptome and Metabolome Reveal Ferulic Acid as a Critical Phenylpropanoid for Drought Resistance in Dendrobium sinense
Source: Plants (Basel). 2025 Jun 15;14(12):1841. doi: 10.3390/plants14121841 (PMC12197015; doi:10.3390/plants14121841)
Supplement: Supplementary file 1 [file plants-14-01841-s001.zip › Supplementary Table S2.pdf]

Table S2 Phenotypes of wild-type and transgenic lines

	Leaf length* (cm)	Leaf width* (cm)	rosette leaf diameter (cm)	leaf number
WT	2.35±1.31a	1.33±0.28a	8.22±1.54a	18.50±2.21a
Line1	2.52±1.29a	1.31±0.22a	8.72±1.33a	19.20±3.24a
Line2	2.49±1.39a	1.30±0.29a	8.52±1.69a	20.27±3.11a
Line3	2.52±1.51a	1.36±0.31a	9.21±1.36a	18.12±3.19a

* The statistics are based on the data of the first true leaf.

Different letters indicate significant statistical differences ($P < 0.05$).
